# Tuning Multi-Wavelength Reflection Properties of Porous Silicon Bragg Reflectors Using Silver-Nanoparticle-Assisted Electrochemical Etching

**DOI:** 10.3390/mi16111198

**Published:** 2025-10-22

**Authors:** Sheng-Yang Huang, Hsiao-Han Hsu, Amal Muhammed Musthafa, I-An Lin, Chia-Man Chou, Vincent K. S. Hsiao

**Affiliations:** 1 Department of Surgery, Taichung Veterans General Hospital, Taichung 407219, Taiwan; drugholic@vghtc.gov.tw; 2 Department of Applied Materials and Optoelectronic Engineering, National Chi Nan University, Nantou 54561, Taiwan; s112328011@mail1.ncnu.edu.tw (H.-H.H.); s112328006@mail1.ncnu.edu.tw (I.-A.L.); 3 Centre for Education (CFE), CSIR-Central Electrochemical Research Institute (CECRI), Karaikudi 630003, Tamil Nadu, India; s113328403@ncnu.edu.tw; 4 Department of Post-Baccalaureate Medicine, College of Medicine, National Chung Hsing University, Taichung 40227, Taiwan

**Keywords:** porous silicon, Bragg reflector, silver nanoparticles, electrochemical etching, multi-wavelength reflection, one-dimensional periodic structure

## Abstract

This study proposes an innovative silver-nanoparticle-assisted electrochemical etching method for the fabrication of porous silicon Bragg reflectors with multi-wavelength reflection characteristics. By introducing silver nanoparticles at varying concentrations (0.1–10 mg/mL) into the conventional HF–ethanol electrolyte and applying periodically modulated current densities (40/100 mA/cm^2^), the transition from single-peak to multi-peak reflection spectra was successfully achieved. The results demonstrate that at a concentration of 10 mg/mL silver nanoparticles, up to four distinct reflection bands can be obtained. A systematic investigation was conducted on the influence of etching cycles (4–20 cycles) and silver nanoparticle concentration on the optical performance and microstructure. SEM analysis revealed well-defined periodic multilayer structures, while XPS analysis confirmed the presence of metallic silver on the porous silicon surface. This work provides a simple, controllable, and cost-effective approach to the development of multifunctional photonic devices, with promising applications in laser optics, solar cells, chemical sensing, and surface-enhanced Raman scattering.

## 1. Introduction

Porous silicon (PSi) Bragg reflectors, as one-dimensional photonic crystal structures, have demonstrated remarkable potential in modern photonics and electrochemistry [1,2,3]. Since the discovery of PSi’s ability to form high-quality optical dielectric layers [4], electrochemical etching has emerged as a core technique for the fabrication of advanced photonic devices. The introduction of current density modulation [5,6] established the theoretical foundation for creating multilayer structures with tailored refractive index distributions through precise control during anodization. Such a high index contrast enables PSi reflectors to achieve broadband, high reflectivity with a relatively small number of layers. Electrochemical etching of silicon is inherently self-limiting, with pore formation controlled by current density and a predictable link between porosity and refractive index [7]. In laser optics, PSi reflectors have been successfully implemented in Ti:Sapphire laser systems, achieving reflectivities above 98% in the 730–1000 nm range and generating 80 fs pulses in mode-locked operation [8]. In photovoltaics, PSi Bragg reflectors have enhanced short-circuit current and efficiency in thin-film silicon solar cells by up to 12%, with a sevenfold optical path enhancement factor [9]. Both numerical simulations and experimental studies have further verified the excellent performance of PSi reflectors on monocrystalline and polycrystalline silicon substrates [10,11,12]. In chemical sensing, PSi reflector-based sensors are highly attractive due to their large surface-to-volume ratio and tunable optical properties [13]. These structures enable effective detection of analytes by altering the effective refractive index through molecular adsorption within the pores, producing measurable optical signals. In solution sensing, PSi Bragg reflectors and microcavities have been employed for the detection of liquids with different refractive indices, exhibiting excellent sensitivity and selectivity [14]. In gas sensing, the porous architecture provides abundant adsorption sites for gas molecules, allowing efficient detection of volatile organic compounds and environmental gases [15,16]. Furthermore, the biocompatibility and surface functionalization capability of PSi enable promising applications in biosensing, including the detection of proteins, DNA, and cells [17]. These diverse sensing functionalities position PSi as an ideal material for multifunctional sensing platforms, offering rapid and accurate analysis of complex samples. Surface-enhanced Raman scattering (SERS) represents another breakthrough that further broadens the application scope of PSi Bragg reflectors [18]. Silver-nanoparticle-decorated PSi microcavity substrates, combining localized surface plasmon resonance with photonic resonance, have achieved low detection limits for methyl parathion [19]. Despite the outstanding performance of PSi Bragg reflectors across diverse applications, existing studies have largely focused on optimizing for single function, with limited understanding of the systematic correlation between electrochemical preparation parameters and multifunctional performance. Moreover, achieving precise control of electrochemical processing parameters to design and fabricate multifunctional devices remains a critical challenge.

Conventional PSi Bragg reflectors typically exhibit single-wavelength or narrow-band reflection. While advantageous in certain contexts, this characteristic poses significant limitations in applications requiring multi-wavelength reflection or broadband regulation. Spectral tuning has traditionally been achieved by adjusting electrochemical etching parameters, such as current density, etching time, and electrolyte composition [7,20,21,22,23]. However, these approaches face technical bottlenecks in realizing multi-wavelength reflection. To overcome such limitations, a variety of innovative spectral modulation techniques have been explored. Radiation processing, for instance, has demonstrated that exposing silicon substrates to γ-rays or ion beams prior to anodization enables precise tuning of distributed Bragg reflector reflection wavelengths [24]. γ-ray irradiation introduces uniformly distributed, low-density defects throughout the sample, while ion irradiation generates high-density localized defects at controlled depths. Further development of low-energy ion irradiation has revealed unique advantages. For example, Ar ions irradiation does not alter the crystalline structure of PSi but effectively disrupts surface bonds and modifies surface states [25]. Reactive ion irradiation represents another major advance. Unlike inert gas ions, high-energy oxygen ions not only induce physical bombardment but also promote chemical reactivity, enabling controlled oxidation of the silicon matrix [26]. Although PSi Bragg reflectors exhibit excellent performance in various applications, conventional electrochemical etching remains limited, typically producing only single-wavelength or narrow-band reflectors. A major challenge may lie in developing a universal fabrication method that meets the diverse demands of lasers, sensors, and SERS substrates.

To address these limitations, the present study introduces an innovative silver-nanoparticle-assisted electrochemical etching method for fabricating PSi Bragg reflectors. Silver nanoparticles (Ag NPs), owing to their excellent electrical conductivity, are expected to alter current distribution and local electric field patterns when uniformly dispersed in the etching environment, thereby influencing pore formation kinetics. Our results reveal that reflectors etched without Ag NPs exhibit only a single broadband reflection. In contrast, with increasing Ag NP concentration, the reflection spectrum evolves to exhibit multiple peaks, reaching up to four distinct bands at a concentration of 10 mg/mL Ag NPs. By incorporating varying concentrations of silver nanoparticles into the conventional HF–ethanol electrolyte, this study demonstrates precise modulation of PSi Bragg reflector optical properties. This strategy not only enables a novel transition from single-wavelength to multi-wavelength reflection through nanoparticle-modified etching kinetics but also offers enhanced control over the optical response of the porous architecture. Compared with existing approaches, this method offers simplified processing, reduced cost, high reproducibility, and strong scalability. The findings are expected to provide a new technological pathway for the design and fabrication of multifunctional PSi photonic devices, paving the way toward industrial-scale applications in multifunctional optics.

## 2. Materials and Methods

**Figure 1:**
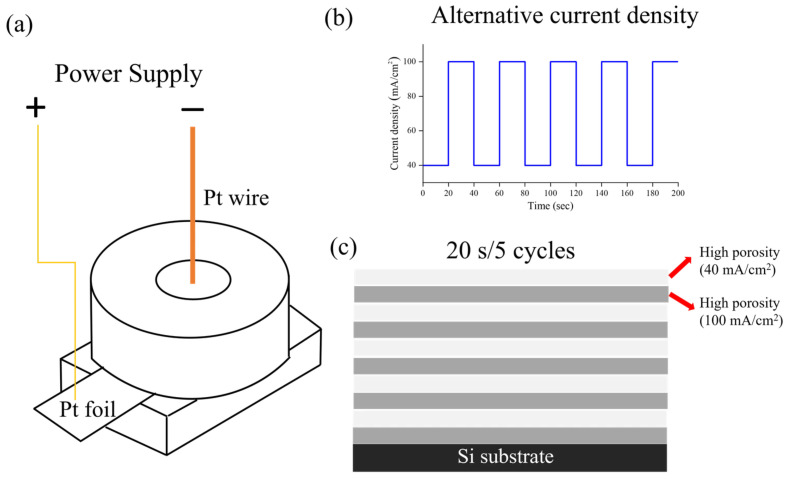
(**a**) Schematic of electrochemical etching cell. (**b**) Current density modulation profile showing the square-wave alternation between 40 and 100 mA/cm^2^ with time intervals corresponding to the desired layer thickness. (**c**) Cross-sectional schematic of the resulting multilayer structure showing alternating high-porosity layers (lighter, formed at 40 mA/cm^2^ with larger pores) and low-porosity layers (darker, formed at 100 mA/cm^2^ with smaller pores).

P-type silicon wafers with a (111) orientation and a resistivity of 0.004–0.006 Ω·cm were used as the substrates for porous silicon formation. The wafers were cut into 2 × 2 cm^2^ samples and cleaned prior to electrochemical etching. AgNPs with an average diameter of 30 nm were employed. Hydrofluoric acid (HF, 48% aqueous solution, electronic grade) and anhydrous ethanol (99.9%) were used as received without further purification. Before electrochemical etching, the silicon substrates were subjected to a standard cleaning procedure to remove organic contaminants and the native oxide layer. The samples were ultrasonically cleaned in acetone for 10 min, followed by ethanol for another 10 min. After rinsing with deionized water and drying under a nitrogen stream, the wafers were immersed in 5% HF solution for 2 min to remove the native oxide and were immediately transferred into the electrochemical cell for etching. The anodization process was carried out in a custom-designed two-electrode electrochemical cell made of polytetrafluoroethylene (PTFE) [27]. The silicon wafer served as the anode, while a platinum wire electrode was used as the cathode. An O-ring sealed the etching area, defining a circular exposed region with a diameter of ~1.2 cm, corresponding to an effective etching area of ~1.13 cm^2^. The cell was connected to a programmable current source capable of precise modulation of current density. The etching electrolyte was prepared by mixing silver nanoparticle aqueous dispersion, anhydrous ethanol, and 48% HF solution in a volume ratio of 1:1:1. Different concentrations of Ag NPs ranging from 0 to 10 mg/mL were investigated. The electrolyte was freshly prepared before each experiment and maintained at room temperature during etching. PSi-BRs were fabricated by anodization under modulated current density, generating periodic variations in porosity and thus alternating high- and low-refractive-index layers. For all samples, the total etching duration was fixed at 200 s. High-porosity layers were formed at a current density of 40 mA/cm^2^, whereas low-porosity layers were obtained at 100 mA/cm^2^. The switching of current density was precisely controlled by the computer-programmed current source to ensure reproducibility. After etching, the samples were thoroughly rinsed with deionized water to remove residual HF and electrolytes and then dried under a nitrogen stream at room temperature. Figure 1 shows a schematic illustration of the Ag-NP-assisted electrochemical etching process and the resulting multilayer structure. The alternating current density modulation (40 mA/cm^2^ for high-porosity layers and 100 mA/cm^2^ for low-porosity layers) creates periodic variations in pore size and density, forming a one-dimensional photonic crystal structure. Silver nanoparticles dispersed in the electrolyte interact with the silicon surface during etching, modifying the local electrochemical environment and enhancing the porosity contrast between alternating layers. The dried PSi-BRs were immediately characterized to minimize oxidation from air exposure. The optical properties of the PSi-BRs were evaluated using reflectance spectroscopy using an Ocean Optics HR4000 high-resolution spectrometer in reflection mode, covering the 400–1000 nm range. A halogen lamp was employed as the light source, with normal incidence geometry. A standard white reference plate was used for calibration, and the reflectance was defined as the ratio of the reflected intensity from the sample to that of the reference. The morphology and microstructure of the samples were examined through scanning electron microscopy (SEM). Alternating high- and low-porosity layers were clearly observed, and their thickness and pore size distribution were analyzed from cross-sectional images. The chemical composition and elemental distribution of the samples were determined through X-ray photoelectron spectroscopy (XPS). XPS analysis was used to confirm the presence and chemical state of silver nanoparticles within the porous silicon structure, as well as the chemical states of silicon and oxygen. The measurements were conducted without additional surface treatment. The positions and intensities of Ag 3d, Si 2p, and O 1s peaks were analyzed to evaluate the chemical state of AgNPs and the degree of surface oxidation.

## 3. Results and Discussion

### 3.1. Effect of Silver Nanoparticles on the Spectral Properties of Porous Silicon Bragg Reflectors

**Figure 2:**
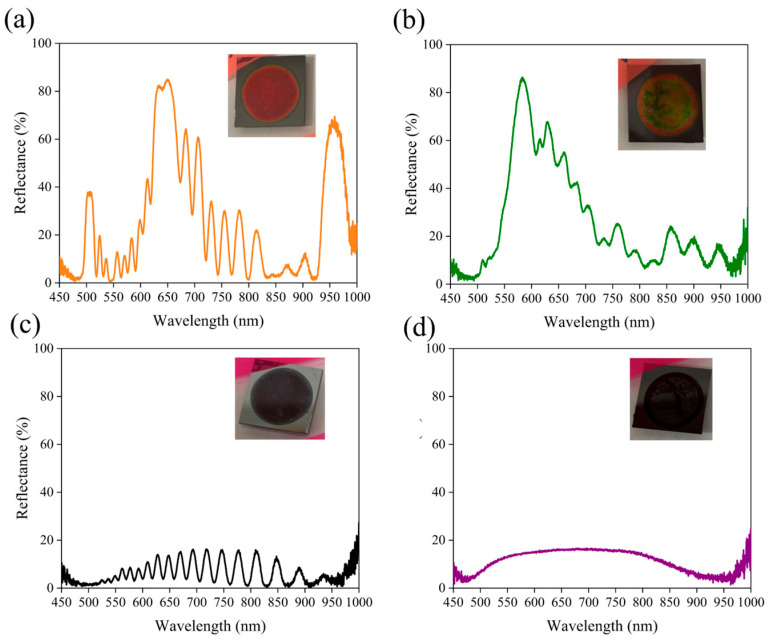
Reflectance spectra and images (inset) of PSi-BRs fabricated under different etching conditions with and without Ag NPs. (**a**) PSBR with 5 mg/mL Ag NPs (10 s/10 cycles), showing multiple peaks. (**b**) Control sample without Ag NPs, showing a single broad peak. (**c**) Sample etched at constant 40 mA/cm^2^, lacking Bragg features. (**d**) Sample etched at constant 100 mA/cm^2^, also lacking Bragg features.

Figure 2a,b demonstrate the pronounced influence of Ag NPs on the reflectance spectra of PSi-BRs. Under identical electrochemical etching conditions, the control sample without Ag NPs (Figure 2b) exhibited a typical single broad reflectance peak. The peak was centered at ~600 nm with a maximum reflectance close to 90% and a full width at half maximum (FWHM) of ~100 nm. This single-peak response is characteristic of conventional PSi-BR, reflecting constructive interference within the periodic multilayer structure. The optical image of the sample revealed a uniform yellow-green appearance, consistent with its selective reflectance in the visible region. In contrast, when 5 mg/mL of Ag NPs were added into the etching electrolyte (Figure 2a), the reflectance spectrum of the PSi-BR changed dramatically. Instead of a single broadband peak, multiple distinct reflectance peaks appeared. A strong primary peak emerged around 650 nm with reflectance exceeding 80%, accompanied by a secondary peak at ~950 nm with ~65% reflectance. Furthermore, several medium-intensity peaks were observed in the intermediate wavelength range of 700–850 nm, forming a complex multi-peak structure. This transition from single- to multi-peak spectra confirms that the presence of Ag NPs significantly alters both the etching dynamics and the resulting optical response. The corresponding sample exhibited a reddish color, in agreement with the enhanced reflectance in the longer-wavelength region. The emergence of multi-peak reflectance can be attributed to the involvement of Ag NPs, though the exact mechanism cannot be fully resolved with the present characterization tools. A plausible explanation is that the excellent conductivity of Ag NPs modifies the current distribution and local electric field within the electrochemical cell. Suspended NPs may act as nanoscale electrodes (resembling a localized metal-assisted chemical etching process), creating additional charge transport pathways during etching and thereby enhancing spatial heterogeneity in pore formation under modulated current densities. To further clarify the role of Ag NPs, Figure 2c,d present control experiments under fixed current density conditions. These are essential for confirming the necessity of periodic current modulation in forming Bragg structures. Figure 2c shows the reflectance spectrum of a sample etched for 200 s at a constant low current density of 40 mA/cm^2^ in the presence of 5 mg/mL Ag NPs. Unlike the clear multi-peak spectrum in Figure 2a, this sample displayed a relatively flat spectrum across the measured range, with low reflectance (10–15%) and only weak oscillations. Similarly, Figure 2d shows the spectrum of a sample etched under a constant high current density of 100 mA/cm^2^ with 5 mg/mL AgNPs. Again, no effective Bragg reflection was observed; instead, the spectrum remained broadly flat with ~15% reflectance across the entire wavelength range. Compared to the low-current-density sample, the spectrum here was smoother, with almost no oscillations. The corresponding sample also appeared black, indicating that light was strongly scattered or absorbed within the porous structure rather than selectively reflected. These control experiments demonstrate that the presence of Ag NPs alone is insufficient to induce multi-peak reflection. Periodic modulation of current density remains essential for establishing the Bragg structure, while Ag NPs serve as auxiliary agents that modulate local etching kinetics and fine-tune the optical properties.

### 3.2. Cross-Sectional Analysis of Porous Silicon Bragg Reflectors

**Figure 3:**
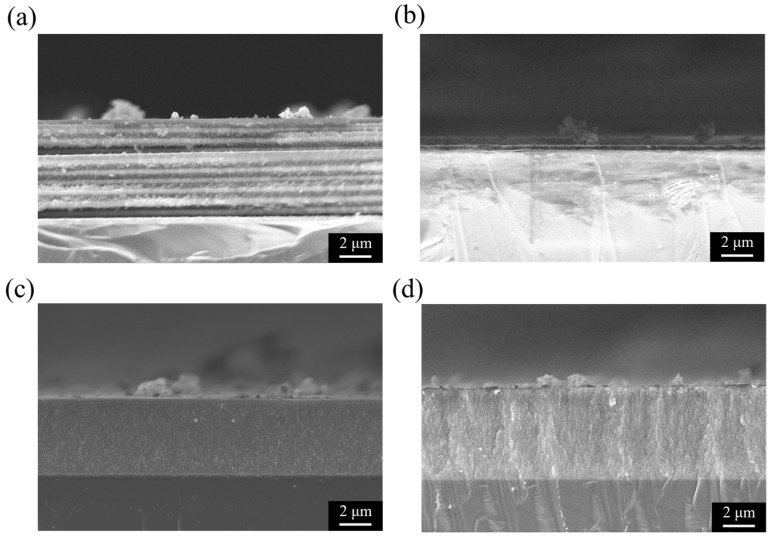
Cross-sectional SEM images of PSi structures under different etching conditions. (**a**) PSi-BR prepared with 5 mg/mL AgNPs under 10 s/10 cycles of current density modulation (40/100 mA/cm^2^), showing well-defined multilayer periodicity. (**b**) Control sample without Ag NPs under the same modulation, showing weaker contrast between layers. (**c**) Sample etched at a constant current density of 40 mA/cm^2^ for 200 s, showing a uniform single-layer structure without periodicity. (**d**) Sample etched at a constant current density of 100 mA/cm^2^ for 200 s, forming a thicker porous layer with vertically aligned pores but no periodic modulation.

Figure 3 shows the cross-sectional SEM images of PSi structures prepared under different etching conditions, directly revealing the influence of Ag NPs and current density modulation on the resulting morphology. These structural observations are consistent with the reflectance spectra discussed earlier and provide direct evidence for the correlation between optical performance and microstructure. As shown in Figure 3a, a PSi-BR fabricated with 5 mg/mL Ag NPs under 10 s/10 cycles of current density modulation (40/100 mA/cm^2^) exhibits a well-defined multilayer structure with about ten alternating high- and low-porosity layers. The contrast between layers is pronounced, with brighter regions corresponding to higher porosity and larger pore sizes, and darker regions corresponding to denser, lower-porosity layers [22]. In comparison, the control sample without Ag NPs (Figure 3b) also shows slightly periodic layering under the same modulation, but with weaker contrast and smoother interfaces. This suggests that Ag NPs enhance the porosity contrast between high and low current density phases, thereby sharpening the multilayer structure, while having little effect on the overall etching rate. In contrast, samples etched under constant current density show no periodic modulation. At 40 mA/cm^2^ (Figure 3c), the porous layer is relatively thin (6–7 μm) and homogeneous, with uniform contrast throughout the depth. At 100 mA/cm^2^ (Figure 3d), the porous layer is thicker (12–14 μm) and features vertically aligned pores extending into the substrate, but again without periodic modulation. These single-layer structures correspond to the weak reflectance observed in Figure 2c,d, confirming that periodic current density modulation is essential for Bragg reflector formation. In all cases, the porous layers remain well adhered to the silicon substrate, without cracks or delamination, indicating good controllability of the electrochemical etching process. The total layer thickness scales with etching current and time as expected, with the constant high-current sample being the thickest, the constant low-current sample the thinnest, and the modulated samples lying in between.

### 3.3. Influence of Etching Conditions on Surface Oxidation Revealed by XPS

**Figure 4:**
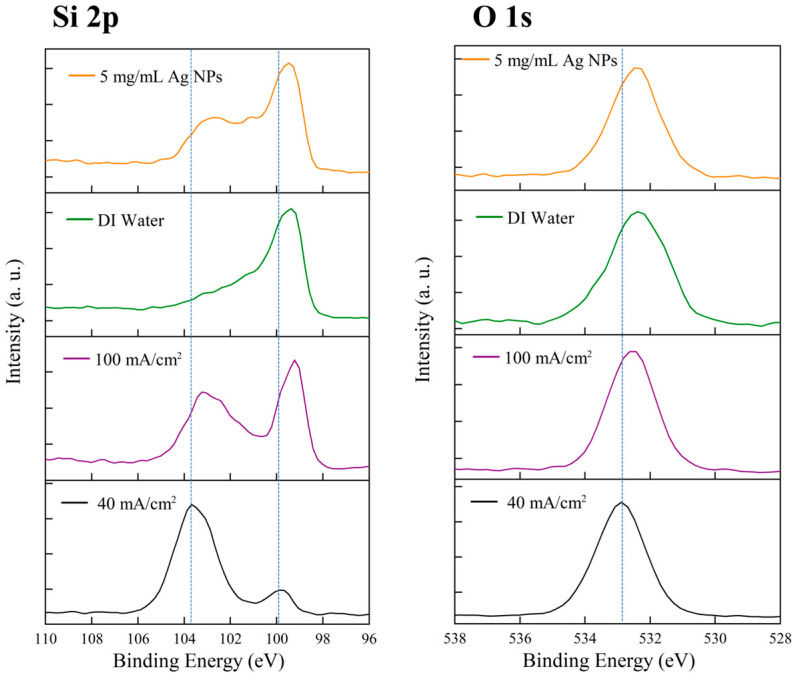
XPS spectra of porous silicon samples under different fabrication conditions. **Left**: Si 2p spectra; **Right**: O 1s spectra. Orange: 5 mg/mL Ag NPs, 10 s/10 cycle modulation; Green: control (DI water), 10 s/10 cycle modulation; Purple: 5 mg/mL Ag NPs, 100 mA cm^−2^ constant etching (200 s); Black: 5 mg/mL Ag NPs, 40 mA cm^−2^ constant etching (200 s).

Figure 4 shows the XPS results of PSi samples prepared under different conditions, including the Si 2p and O 1s core-level spectra. These chemical analyses reveal the oxidation states of the PSi surfaces and the influence of fabrication conditions on surface composition. In the Si 2p spectra (left panel), all samples exhibit characteristic peaks near 99–100 eV and 103–104 eV. The peak at 99–100 eV corresponds to the Si 2p signal of elemental silicon, representing the unoxidized Si framework, while the 103–104 eV peak is attributed to silicon in SiO_2_, reflecting the degree of surface oxidation. The relative intensity of these two peaks varies among samples, providing insight into their oxidation levels. For the sample prepared with 5 mg/mL AgNPs under 10 s/10-cycle current density modulation (orange curve), the Si 2p spectrum displays a clear double-peak structure, with a dominant peak at ~99–100 eV (elemental Si) and a shoulder at ~103 eV (SiO_2_). This indicates the coexistence of an unoxidized silicon backbone and a partially oxidized surface layer. The oxidation component likely originates from natural oxidation upon air exposure after etching or from partial oxidation during the electrochemical process. The control sample without Ag NPs (green curve, labeled DI Water), fabricated under the same modulation conditions, shows a main peak at ~99–100 eV with a weaker shoulder around 103–104 eV. Compared with the Ag NP-assisted sample, the oxidation-related peak is slightly less pronounced, suggesting that the presence of Ag NPs may influence surface oxidation to some extent. The sample etched continuously at a fixed current density of 100 mA/cm^2^ (purple curve) exhibits a more prominent double-peak feature, with a strong SiO_2_ peak near 103 eV and a main Si peak near 99–100 eV. The higher relative intensity of the oxide peak suggests a greater degree of surface oxidation under high current density, possibly due to more intense electrochemical reactions and a larger effective surface area. Conversely, the sample etched continuously at 40 mA/cm^2^ (black curve) shows a main Si peak at ~99–100 eV with only a weak shoulder around 103–104 eV, indicating a lower oxidation level. This may be related to slower etching kinetics and structural differences in the pores formed under low current density.

The O 1s spectra (right panel) provide complementary information about the oxidation state. All samples show a clear O 1s peak centered around 533 eV, characteristic of oxygen in SiO_2_, confirming the presence of surface oxides consistent with the Si 2p results. The sample with 5 mg/mL Ag NPs (orange curve) exhibits a relatively strong, symmetric O 1s peak at ~533 eV. The control DI Water sample (green curve) also shows a similar peak at 533 eV, with comparable intensity, suggesting a similar surface oxygen content. Samples etched at 100 mA/cm^2^ (purple) and 40 mA/cm^2^ (black) show O 1s peaks of similar intensity and position. Overall, the O 1s results indicate that all samples underwent comparable levels of surface oxidation, which likely occurred during post-etching air exposure rather than during the etching process itself. The consistent binding energy (~533 eV) across all samples suggests a uniform chemical environment for oxygen, predominantly in the form of SiO_2_.

### 3.4. Influence of Etching Cycles on the Spectral Characteristics of Porous Silicon Bragg Reflectors

**Figure 5:**
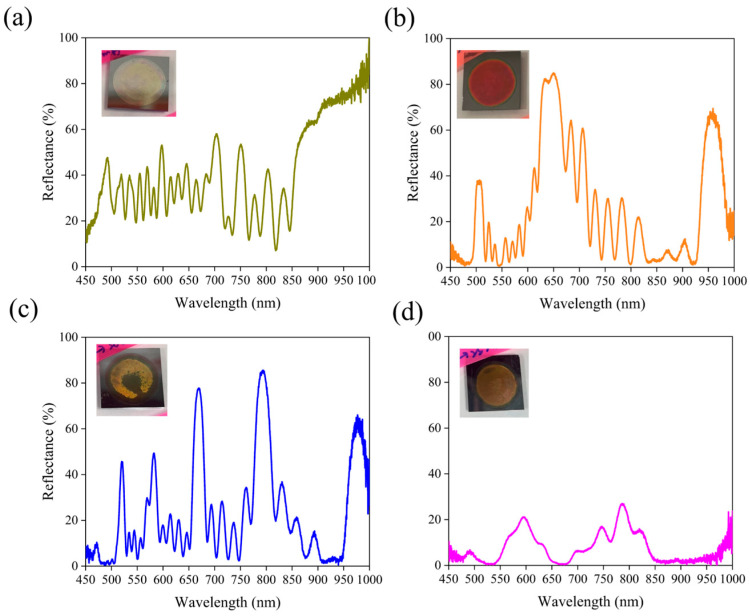
Reflection spectra of PSi-BRs fabricated at a fixed Ag NP concentration of 5 mg/mL under different etching cycle conditions. All samples were etched with alternating current densities of 40 and 100 mA/cm^2^ for a total duration of 200 s. (**a**) 5 s/20 cycles; (**b**) 10 s/10 cycles; (**c**) 20 s/5 cycles; (**d**) 25 s/4 cycles. Insets show the corresponding optical photographs of the samples.

Figure 5 illustrates the significant influence of varying the number of etching cycles and the etching time per layer on the reflection spectra of PSi-BRs, under a fixed Ag NP concentration of 5 mg/mL. All samples were prepared with a total etching time of 200 s, while the switching frequency between high and low current densities was adjusted to form structures with different periodicities and layer thickness distributions. Figure 5a shows the reflection spectrum of the sample fabricated under the high and low current densities alternated every 5 s for 20 repetitions, forming 20 pairs of thin layers. The spectrum exhibits complex oscillatory features across 450–850 nm, with reflectance fluctuating between 20–50% and several moderate reflection peaks. A pronounced increase in reflectance appears in the near-infrared region above 850 nm, reaching approximately 80–90% around 950–1000 nm. The sample shows a light yellowish-white appearance, consistent with its relatively low and dispersed reflectance in the visible region and strong reflection in the near-infrared. These spectral characteristics indicate that the structure formed by thinner layers (5 s etching time) and a higher number of periods (20 pairs) exhibits a primary Bragg reflection band located in the near-infrared region. Figure 5b presents the reflection spectrum of the sample prepared under the 10 s/10-cycle condition, which has been discussed in detail in Figure 2a. The resulting structure displays distinct multi-peak reflection features, with a strong primary reflection peak at 650 nm exceeding 80% reflectance, and an additional peak around 950 nm in the near-infrared region with about 65% reflectance. Several medium-intensity peaks are also observed in the 700–850 nm range. The sample appears reddish, corresponding to its high selective reflectivity in the long-wavelength region of the visible spectrum. The 10 s/10-cycle configuration appears to achieve a favorable balance between layer thickness and number of periods, providing sufficient optical contrast while generating a rich multi-peak structure spanning from visible to near-infrared wavelengths. Figure 5c shows the reflection spectrum of the sample fabricated under the 20 s/5-cycle condition. With a longer etching time per layer, five pairs of relatively thick layers are formed. The spectrum features several sharp and high-intensity reflection peaks, the most prominent located at approximately 650 nm and 750 nm, with reflectance values reaching about 80–85%. Additional peaks can be observed around 500–600 nm and 900–950 nm. Compared with the previous two conditions, these peaks exhibit narrower full widths at half maximum and higher peak reflectance, indicating superior optical quality factors. The sample appears yellowish-green, consistent with its strong multi-peak reflection between 550–750 nm. These results suggest that although the 20 s/5-cycle structure has fewer periods, the thicker layers can still produce an effective Bragg reflection with high peak quality. Figure 5d displays the reflection spectrum of the sample prepared under the 25 s/4-cycle condition, representing the extreme case of the fewest periods and the thickest individual layers for the same total etching time. The spectrum is relatively flat overall, showing only broad and weak modulations around 550 nm, 750 nm, and 850 nm, with a maximum reflectance of about 20–25%. No distinct high-intensity reflection peaks are observed, in sharp contrast to the other conditions. The sample appears brownish, consistent with its generally low and uniform reflectance across the visible spectrum. This result clearly indicates that when the number of periods is reduced to only four pairs, effective Bragg reflection cannot be established even with increased layer thickness. This is likely due to the insufficient periodic repetitions needed to produce strong multiple interference reflections. As the etching time per layer increases from 5 to 25 s and the number of periods decreases from 20 to 4 pairs, the reflection spectra evolve from near-infrared-dominant to multi-peak visible reflection and eventually to weak, broadband reflection. The 5 s/20-cycle structure primarily reflects in the near-infrared region, while the 10 s/10-cycle and 20 s/5-cycle structures exhibit efficient multi-peak reflection across the visible and near-infrared regions. In contrast, the 25 s/4-cycle structure nearly loses its Bragg reflection capability. Moreover, comparing Figure 5b,c provides particularly insightful results. These configurations—10 s/10 cycles and 20 s/5 cycles—have identical total etching times but differ by a factor of two in layer thickness and number of periods. Although both exhibit multiple high-reflectance peaks, the peak positions, numbers, and shapes differ. The 10 s/10-cycle sample displays more dispersed peaks across a broader wavelength range, whereas the 20 s/5-cycle sample shows sharper and more concentrated peaks. This comparison reveals that within the Ag NP-assisted etching system, the interplay between layer thickness and periodicity exerts a subtle yet significant influence on the final spectral characteristics.

### 3.5. Cross-Sectional Morphology Analysis of Porous Silicon Structures with Different Etching Cycles

**Figure 6:**
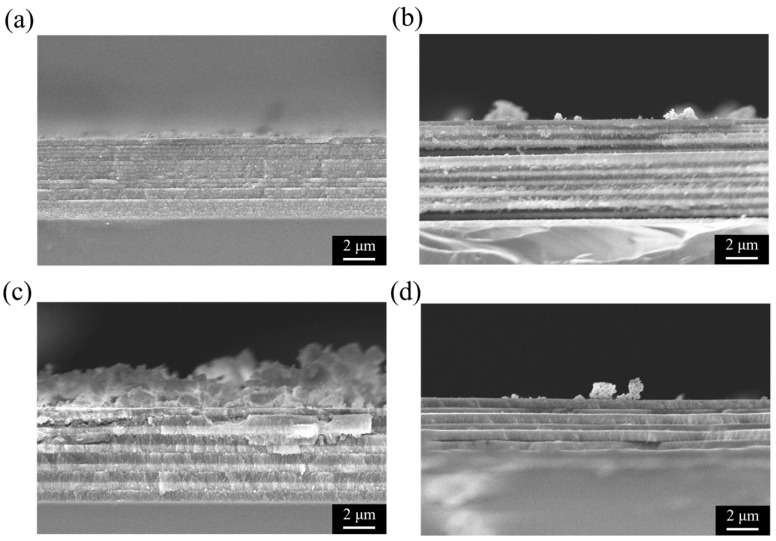
Cross-sectional SEM images of PSi-BRs prepared under different etching cycle conditions: (**a**) 5 s/20 cycles, (**b**) 10 s/10 cycles, (**c**) 20 s/5 cycles, and (**d**) 25 s/4 cycles. The images reveal the evolution of layer thickness and period number under a fixed total etching time of 200 s, showing the trade-off between structural periodicity and layer thickness that governs the optical reflection behavior.

Figure 6 presents the cross-sectional SEM images of the PSi-BRs corresponding to the spectra shown in Figure 5, directly revealing how the etching cycle parameters influence the microscopic structural features of PSi. These images provide critical structural evidence for understanding the correlation between etching cycles and optical performance. Figure 6a shows the cross-section of the sample prepared under the 5 s/20 cycles condition. The image clearly reveals densely stacked multilayers, with approximately 20 alternating pairs of high- and low-porosity layers distinguishable. Since each layer was etched for only 5 s, the individual layer thickness is relatively small, estimated to be about 200–300 nm. The contrast between adjacent layers is visible but relatively soft, indicating that the difference in porosity between the high- and low-porosity layers is less pronounced than in thicker-layer structures. The total porous region thickness is about 8–10 μm, with a smooth surface and minor deposition. This dense thin-layer structure corresponds well with the strong near-infrared reflection observed in Figure 5a, as the numerous thin periodic layers provide efficient multiple interference pathways for near-infrared light. Figure 6b displays the cross-section of the sample etched under the 10 s/10 cycles condition, corresponding to the same sample as shown in Figure 3a. The structure exhibits ten clearly alternating pairs of high- and low-porosity layers with distinct contrast differences. Each layer is noticeably thicker than that in the 5 s/20 cycles sample, with an estimated thickness of 400–600 nm per layer. The high-porosity layers appear brighter with a more open pore network, while the low-porosity layers appear darker and denser. The total film thickness is about 10–12 μm, with granular surface deposits. This configuration with a moderate number of cycles and layer thickness corresponds to the rich multi-peak reflection spectra in Figure 5b, confirming that this parameter set effectively enables multiband reflection across the visible to near-infrared range. Figure 6c presents the sample etched under the 20 s/5 cycles condition. The structure shows five clearly defined thick bilayers, with each layer significantly thicker—approximately 800–1200 nm. Although the number of cycles is lower, the contrast between adjacent layers is very sharp, making the alternation between high- and low-porosity layers easily identifiable. The total porous thickness is about 10–12 μm, comparable to other samples. Notably, the thick-layer structure exhibits good structural integrity with relatively smooth interlayer interfaces. More granular surface deposits are observed, forming irregular textures. This configuration corresponds well to the multiple sharp, high-intensity reflection peaks observed in Figure 5c. Despite having fewer periods, the thicker individual layers still provide sufficient optical path differences to achieve effective Bragg reflection. Figure 6d shows the sample prepared with 25 s/4 cycles, which has the fewest periods among all samples. Only about four pairs of thick layers can be identified, each with an estimated thickness of 1000–1500 nm or more. Although the layer alternation is still visible, the total number of periods is clearly insufficient. The total porous thickness is again about 10–12 μm, like the other samples. The surface shows considerable granular deposition and irregular morphology. This configuration corresponds directly to the low reflectivity spectrum observed in Figure 5d, confirming that when the number of cycles decreases to four, the structure cannot sustain sufficient multiple reflections to form a strong Bragg response, even though each layer is thicker.

A systematic comparison of Figure 6 reveals a clear trade-off between the layer thickness and the number of cycles. Under a fixed total etching time of 200 s, increasing the etching time per layer inevitably reduces the total number of cycles. Structurally, the 5 s/20 cycles sample forms a dense thin-layer array, the 10 s/10 cycles sample forms a balanced intermediate structure, the 20 s/5 cycles sample yields thicker but fewer layers, and the 25 s/4 cycles sample produces the thickest yet sparsely stacked structure. By correlating these structural features with the optical responses, several important conclusions can be drawn. Theoretically, the reflectivity of a Bragg mirror increases with the number of periods; however, each layer’s thickness must satisfy a specific phase-matching condition to produce constructive interference at the target wavelength. The dense thin-layer structure of Figure 6a has many periods but thin layers, shifting the Bragg condition toward the near-infrared region and thus producing high reflection there. The structures in Figure 6b,c strike a balance between layer thickness and number of periods, enabling high reflectivity and multiple reflection bands across the visible–near-infrared range. In contrast, the Figure 6d structure, though it has the thickest layers, suffers from too few periods (only four pairs), leading to insufficient reflection intensity and low overall reflectivity. The combined structural and optical analyses of Figure 5 and Figure 6 clearly establish the relationship between etching cycle parameters, microstructural characteristics, and optical performance. The optimal performance of porous silicon Bragg reflectors requires an appropriate combination of layer thickness and cycle number. Excessively thin multilayers (5 s/20 cycles) shift reflection toward longer wavelengths, moderate configurations (10 s/10 cycles and 20 s/5 cycles) enable broadband multipeak reflection, while insufficient cycle numbers (25 s/4 cycles) result in degraded Bragg reflection efficiency. These structure–performance relationships provide valuable guidance for the design and optimization of Ag NP-assisted PSi photonic devices.

### 3.6. Comparison of Surface Chemical States at Different Etching Cycles

**Figure 7:**
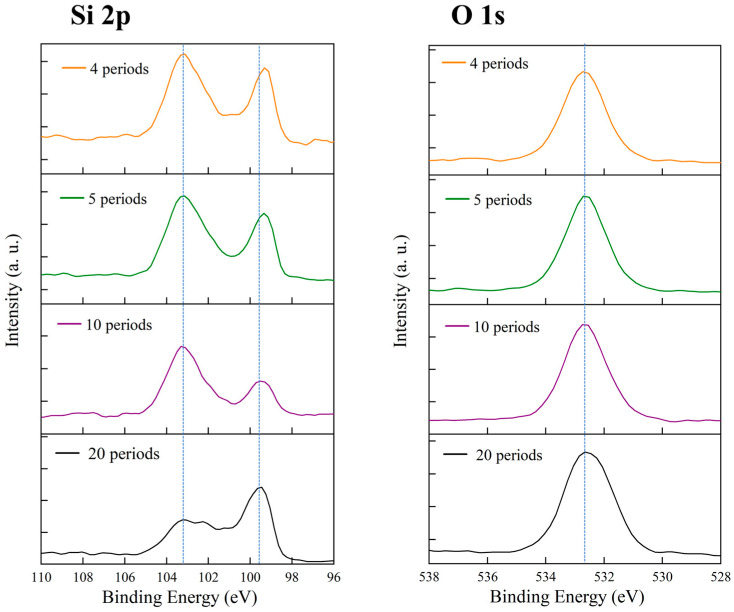
XPS spectra of porous silicon samples under different etching conditions of different periods.

Figure 7 shows the X-ray photoelectron spectroscopy (XPS) analysis of porous silicon samples fabricated with different etching cycle numbers at a fixed Ag NP concentration of 5 mg/mL. The Si 2p and O 1s core-level spectra reveal how the etching periodicity influences surface oxidation behavior. All Si 2p spectra exhibit two characteristic peaks: one at ~99–100 eV corresponding to elemental silicon (Si^0^) and another at ~103–104 eV corresponding to oxidized silicon (Si^4+^). While the peak positions remain similar across samples, their relative intensities vary. The 4-cycle and 5-cycle samples display strong oxide peaks, indicating a higher oxidation degree. The 10-cycle sample shows a relatively weaker oxide component, whereas the 20-cycle sample again exhibits an intensified SiO_2_ peak. This nonmonotonic behavior suggests that surface oxidation is jointly affected by total surface area and pore connectivity under different structural configurations. The O 1s spectra, all centered near 533 eV, confirm the presence of SiO_2_ on the surface. The relative intensity trends of the O 1s peaks are consistent with those of the Si 2p spectra, reinforcing the observed variation in oxidation degree. No noticeable binding-energy shift was detected among the samples, implying that oxygen exists predominantly in a similar SiO_2_ environment. Overall, the results indicate that both extremely low and high etching cycle numbers lead to enhanced surface oxidation, while moderate periodicity (10 cycles) yields the lowest oxide fraction, reflecting a balance between surface exposure and structural compactness.

### 3.7. Effect of Silver Nanoparticle Concentration on the Optical Properties of Porous Silicon Bragg Reflectors

**Figure 8:**
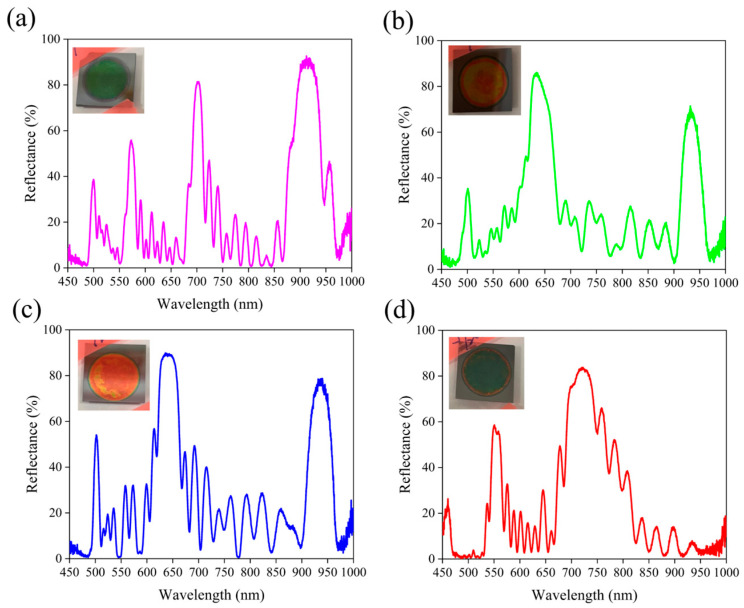
Reflection spectra of PSi-BRs fabricated at a fixed 10 s/10 cycles for a total duration of 200 s under (**a**) 10 mg/mL, (**b**) 2.5 mg/mL, (**c**) 0.5 mg/mL, and (**d**) 0.1 mg/mL Ag NP concentrations. Insets show the corresponding optical photographs of the samples.

Figure 8 presents the reflection spectra of PSi-BRs prepared under fixed etching conditions (10 s/10 cycles) with varying AgNP concentrations from 0.1 to 10 mg/mL. The results reveal a strong concentration-dependent evolution of optical behavior. At the highest concentration (10 mg/mL, Figure 8a), the spectrum exhibits multiple strong reflection peaks distributed across the visible to near-infrared range, with dominant peaks near 750 and 900 nm (reflectivity up to 90%). This multi-band reflection indicates that a high AgNP density induces complex interference structures within the porous network. For 2.5 mg/mL (Figure 8b), two major peaks around 650 and 900 nm are observed, maintaining high reflectivity (~85% and ~70%). The number of reflection bands decreases compared to the 10 mg/mL sample, but the spectral peaks remain sharp and intense, suggesting an optimal balance between periodicity and modulation depth. At 0.5 mg/mL (Figure 8c), the spectrum shows two pronounced peaks at ~620 and 920 nm, with the highest reflectivity (~90%) among all samples. This concentration appears to produce the most uniform and well-defined multilayer structure, yielding high optical quality. When the concentration is further reduced to 0.1 mg/mL (Figure 8d), the reflection remains double-peaked but with weaker intensity (~80%) and broader features, indicating reduced modulation during etching. Overall, increasing the AgNP concentration transforms the reflection spectrum from a simple dual-band pattern into a complex multi-band response. Higher Ag NP concentrations might provide more conductive sites during electrochemical etching, modulating the local current density and inducing variations in porosity and layer thickness. Moderate concentrations generate well-defined alternating layers that enhance constructive interference, whereas excessive Ag NP loading (10 mg/mL) introduces excessive disorder, broadening the reflection bands and slightly reducing the peak reflectivity. These results confirm that controlled Ag NP-assisted etching can effectively tailor the optical response of porous silicon from single- to multi-wavelength reflection by tuning nanoparticle concentration.

### 3.8. Cross-Sectional Morphology of Porous Silicon Structures with Different Silver Nanoparticle Concentrations

**Figure 9:**
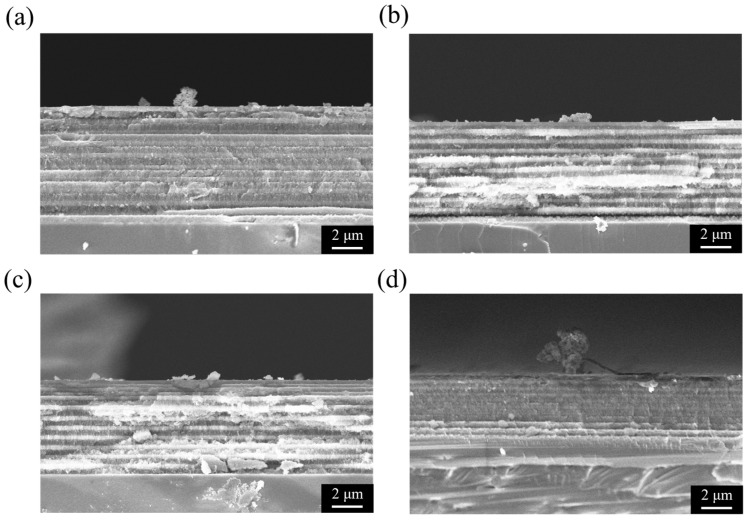
Cross-sectional SEM images of PSi-BRs prepared under (**a**) 10 mg/mL, (**b**) 2.5 mg/mL, (**c**) 0.5 mg/mL, and (**d**) 0.1 mg/mL Ag NP concentrations.

Figure 9a shows the cross-section of the sample prepared with the highest AgNP concentration of 10 mg/mL. The image clearly displays approximately 10 pairs of alternating high- and low-porosity layers, with the layered structure sharply distinguished by contrast. The high-porosity layers appear brighter, while the low-porosity layers appear darker. The total porous layer thickness is about 10–12 μm. Despite the increased surface roughness, the multilayer structure remains well-defined and periodic. Figure 9b presents the cross-section of the sample prepared with 2.5 mg/mL Ag NPs. This sample also exhibits approximately 10 pairs of alternating layers with clear contrast and a total thickness of 8–10 μm, comparable to that of the 10 mg/mL sample. The periodicity and definition of the layered structure are excellent, corresponding to the strong dual-peak reflection spectrum observed in Figure 8b. Figure 9c shows the structure of the sample prepared with 0.5 mg/mL Ag NPs. The image reveals a distinct multilayer configuration with around 10 pairs of high- and low-porosity layers, similar as the condition of using 2.5 mg/mL Ag NPs. The interlayer contrast remains strong, and the periodic features are clearly visible. Interestingly, despite the lower Ag NP concentration, the clarity of the layered structure is not compromised—consistent with the high-quality dual-peak reflection spectrum in Figure 8c. Figure 9d displays the cross-section of the sample prepared with the lowest Ag NP concentration of 0.1 mg/mL. A layered structure can still be observed, though the total porous layer thickness appears slightly reduced. To quantitatively assess the optical quality of the multilayer structures, we calculated the Quality Factor (Q-factor), defined as Q = λ/Δλ, where λ is the central wavelength of the reflection peak and Δλ is the full width at half maximum (FWHM). From the reflection spectra in Figure 7, the 0.5 mg/mL AgNP sample exhibits a dominant reflection peak at 620 nm with FWHM ≈ 28 nm, yielding Q ≈ 22. The 2.5 mg/mL sample shows Q ≈ 20 for the 650 nm peak (FWHM ≈ 33 nm). For the 10 mg/mL sample with multiple peaks, individual Q-factors range from 15–18, indicating broader spectral features. These Q-values are reasonable for 10-bilayer Bragg reflectors and are consistent with values reported for similar electrochemically etched porous silicon structures. The moderate Q-factors reflect the trade-off between achieving multi-wavelength response and maintaining narrow linewidths. Higher Q-factors could potentially be achieved by increasing the number of periods or further optimizing the porosity contrast, though this may limit the multi-band capability that is the key innovation of this work. At the interface between the porous layer and the silicon substrate, a wavy morphology can be observed, possibly indicating minor etching nonuniformities. While the layer structure is still present, its contrast is somewhat lower than that of higher-concentration samples. It is worth emphasizing that all samples contain approximately 10 layer pairs, confirming that the Ag NP concentration does not alter the fundamental periodicity imposed by current-density modulation. The role of Ag NPs is to fine-tune the local etching dynamics within this established periodic framework—modifying layer thickness distribution, porosity gradients, and interfacial properties, thereby influencing the resulting optical behavior. This conclusion aligns with previous findings: Ag NPs act as an auxiliary agent, whose effectiveness relies entirely on a properly designed electrochemical etching strategy. All samples maintain good adhesion to the silicon substrate, and no delamination or structural collapse is observed, demonstrating the process stability of Ag NP-assisted electrochemical etching across the entire concentration range. This stability is crucial for ensuring reliable fabrication in practical applications.

### 3.9. Surface Chemical Composition Analysis of Samples with Different Silver Nanoparticle Concentrations

**Figure 10:**
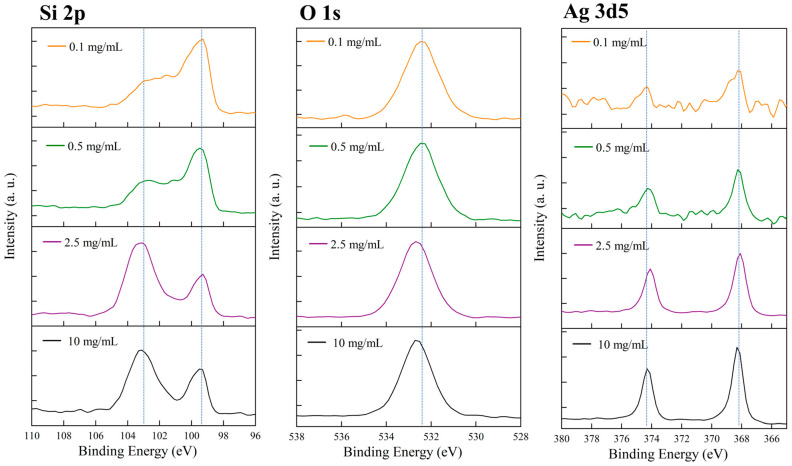
XPS spectra of porous silicon samples under different etching conditions using different Ag NPs.

Figure 10 shows the full XPS analysis of porous silicon samples prepared with different Ag NP concentrations, including the Si 2p, O 1s, and Ag 3d_5_ core-level spectra. The Si 2p spectra exhibit the typical double-peak characteristics in all samples. For the 0.1 mg/mL sample (orange curve), two distinct peaks appear at approximately 103 eV and 99–100 eV, corresponding to SiO_2_ and elemental Si, respectively, with comparable intensities. The 0.5 mg/mL sample (green curve) shows a similar dual-peak structure, though the oxide peak is slightly more intense. The 2.5 mg/mL sample (purple curve) exhibits the same doublet, with Si^0^ and Si^4+^ peaks clearly separated around 99–100 eV and 103 eV. The 10 mg/mL sample (black curve) also presents well-defined dual peaks for elemental silicon and silicon oxide. Comparing the Si 2p spectra across concentrations reveals a minor variation in the relative intensity ratio of SiO_2_ to Si^0^ peaks as Ag NP concentration increases. Higher-concentration samples (2.5 and 10 mg/mL) exhibit slightly stronger SiO_2_ signals, likely due to increased surface complexity and oxidation opportunities arising from enhanced Ag NP deposition. However, the Si 2p peak positions remain essentially constant among all samples, indicating that the fundamental chemical state of silicon is preserved regardless of Ag NP concentration. The O 1s spectra show a pronounced peak at approximately 533 eV in all samples, corresponding to oxygen in SiO_2_. The 0.1 mg/mL sample shows a relatively strong O 1s peak, and the 0.5 mg/mL sample displays a comparable intensity. The 2.5 mg/mL sample exhibits a slightly weaker O 1s signal, while the 10 mg/mL sample shows similar intensity to the medium-concentration samples. All peaks are centered near 533 eV with symmetric shapes, confirming that oxygen predominantly exists in the form of silicon dioxide. The most crucial information is provided by the Ag 3d_5_ spectra, which directly confirm the presence of silver on the porous silicon surfaces. The Ag 3d_5_/_2_ peak located near 368 eV is characteristic of metallic Ag. In the 0.1 mg/mL sample (orange curve), a detectable yet weak Ag 3d_5_ signal appears with two peaks near 368 eV and 374 eV, corresponding to the Ag 3d_5_/_2_ and Ag 3d_3_/_2_ spin–orbit components, respectively. Although weak, the clear presence of these peaks confirms that even at low concentration, Ag NPs can deposit or adsorb onto the porous silicon surface during etching. In the 0.5 mg/mL sample (green curve), the Ag 3d_5_ signal intensifies markedly, and the doublet becomes more distinct, indicating increased silver deposition with higher nanoparticle concentration. The 2.5 mg/mL sample (purple curve) shows further enhancement with strong, well-defined peaks. The 10 mg/mL sample (black curve) exhibits the strongest Ag 3d_5_ signal, with the highest peak intensities at 368 eV and 374 eV, reflecting substantial silver accumulation at high concentrations. The systematic concentration-dependent evolution of the Ag 3d_5_ spectra conveys several key insights. First, the Ag signal intensity increases proportionally with Ag NP concentration in the electrolyte, demonstrating that the amount of deposited silver can be effectively controlled via concentration adjustment. Second, all samples show Ag 3d_5_/_2_ peaks near 368 eV, consistent with metallic Ag binding energy, indicating that the deposited silver mainly exists in a metallic state rather than as an oxide. If silver were oxidized into Ag_2_O or AgO, the Ag 3d_5_/_2_ peak would shift toward higher binding energies (≈367.8 eV or 367.4 eV). The observed position near 368 eV confirms the predominance of metallic silver.

## 4. Conclusions

This study successfully developed a Ag-NP-assisted electrochemical etching technique for the controlled fabrication of PSi-BRs with multi-wavelength reflection characteristics. Systematic investigations revealed that periodic current density modulation is essential for forming the alternating high- and low-porosity layers required for Bragg reflection, while fixed-current etching fails to generate effective periodic structures. The introduction of Ag NPs significantly altered the optical spectra, enabling a tunable transition from single-peak to multi-peak reflection as the nanoparticle concentration increased. Structural and XPS analyses confirmed the presence of metallic Ag within the porous network, whose content scales with electrolyte concentration. The enhanced multi-wavelength response arises from the combined effects of Ag-induced local current redistribution during etching and possible coupling between localized surface plasmon resonances and photonic modes within the layered architecture. The demonstrated AgNP-assisted electrochemical etching approach offers versatile control over resonance characteristics through multiple tunable parameters. Wavelength positioning can be tailored by adjusting the etching time per layer, as demonstrated in Figure 5 and Figure 6, where increasing the individual layer thickness from a 5 s to 20 s etching time shifts the primary reflection band from near-infrared toward visible wavelengths, following the Bragg condition λ = 2(n_1_d_1_ + n_2_d_2_). Multi-wavelength capability is achieved through AgNP concentration modulation (Figure 8 and Figure 9), enabling transitions from conventional single-peak to multi-peak reflection, with up to four distinct bands at a 10 mg/mL concentration. Bandwidth control can be accomplished by varying the number of periods and the refractive index contrast between layers—more periods yield narrower, higher-Q resonances, while fewer periods produce broader bands. Spectral coverage is tunable over a wide range (visible to near-infrared) by combining these parameters. This multi-dimensional parameter space makes the technique highly adaptable for diverse applications: narrow-band resonances for optical sensing, broadband reflection for light-trapping in solar cells, and multi-wavelength response for multiplexed detection platforms. Future work could explore dynamic tuning through the infiltration of active materials into the porous network or integration with microfluidic systems for real-time spectral reconfiguration.

## Data Availability

The data presented in this study are available on request from the corresponding author. The data is unavailable due to privacy.
